# Near-Source Risk Functions for Particulate Matter Are Critical When Assessing the Health Benefits of Local Abatement Strategies

**DOI:** 10.3390/ijerph18136847

**Published:** 2021-06-25

**Authors:** David Segersson, Christer Johansson, Bertil Forsberg

**Affiliations:** 1 Swedish Meteorological and Hydrological Institute, 601 76 Norrköping, Sweden; 2 Department of Environmental Science, Stockholm University, 114 19 Stockholm, Sweden; christer.johansson@aces.su.se; 3 Environment and Health Administration, 104 20 Stockholm, Sweden; 4 Department of Public Health and Clinical Medicine, Section of Sustainable Health, Umea University, 901 87 Umeå, Sweden; bertil.forsberg@umu.se

**Keywords:** dispersion modeling, exposure, particulate matter, health impact assessment, abatement strategies, electrification, studded tires, congestion charges

## Abstract

When mortality or other health outcomes attributable to fine particulate matter (PM_2.5_) are estimated, the same exposure–response function (ERF) is usually assumed regardless of the source and composition of the particles, and independently of the spatial resolution applied in the exposure model. While several recent publications indicate that ERFs based on exposure models resolving within-city gradients are steeper per concentration unit (μgm^−3^), the ERF for PM_2.5_ recommended by the World Health Organization does not reflect this observation and is heavily influenced by studies based on between-city exposure estimates. We evaluated the potential health benefits of three air pollution abatement strategies: electrification of light vehicles, reduced use of studded tires, and introduction of congestion charges in Stockholm and Gothenburg, using different ERFs. We demonstrated that using a single ERF for PM_2.5_ likely results in an underestimation of the effect of local measures and may be misleading when evaluating abatement strategies. We also suggest applying ERFs that distinguish between near-source and regional contributions of exposure to PM_2.5_. If separate ERFs are applied for near-source and regional PM_2.5_, congestion charges as well as a reduction of studded tire use are estimated to be associated with a significant reduction in the mortality burden in both Gothenburg and Stockholm. In some scenarios the number of premature deaths is more than 10 times higher using separate ERFs in comparison to using a single ERF irrespective of sources as recommended by the WHO. For electrification, the net change in attributable deaths is small or within the uncertainty range depending on the choice of ERF.

## 1. Introduction

Despite many years of regulations and abatement strategies, air pollution is today considered the single most important environmental health risk in Europe and globally. According to the Global Burden of Disease (GBD) assessment for 2019, ambient levels of PM_2.5_ (particles with an aerodynamic diameter <2.5 μm) cause around 4.1 million deaths per year [1]. The increase in risk with increased exposure is described by the exposure–response function (ERF), where a linear association is usually assumed. Using a different ERF, 8.7 million deaths were attributed solely to the fossil fuel component of PM_2.5_ in 2018 [2].

Influential publications by the World Health Organization (WHO) have stated that there is not enough evidence to recommend different ERFs for different particle sources [3,4]. The Health Risks of Air Pollution In Europe (HRAPIE) report recommended a hazard ratio (HR) of 1.062 per 10 μgm^−3^ of PM_2.5_ for health impact assessments (HIA) of all-cause mortality at age >30 years [4]. This recommendation was based on a meta-study [5], and was heavily influenced by epidemiological studies based on differences in concentrations between different cities.

Higher risk estimates per increase in mass concentration for soot particles (including black carbon) than for PM_2.5_ have before been reported in meta-analyses of published studies [5,6]. These observations suggest strong effects from primary combustion-related particles, e.g., vehicle exhaust particles, per increase in mass concentration. However, the limited number of studies on PM_2.5_ elemental components have produced rather inconclusive results regarding which components are most important for mortality, perhaps because elements such as Fe, Zn, and K may represent different sources in different places [7].

Most HIAs dealing with air pollution levels (including national studies) [8,9] have focused on particles, whereas some local assessments considered ERFs for NO_x_ or NO_2_ as more relevant for traffic emissions than the ERFs for total PM_2.5_ from older studies with between-city comparisons [10,11,12]. Older epidemiological studies on the associations between total PM exposure and mortality did not provide any alternatives. In some HIAs the authors were concerned about the choice of one single exposure–response function for both the regional background exposure and the contribution of PM from local sources, and stressed the need for studies with a higher spatial resolution [8]. In other assessments it was concluded that the application of ERFs from studies using central monitors results in an underestimation of health impacts [13].

A HIA or a cost–benefit analysis based on a regional HR that severely underestimates the benefits of reducing local emissions would discourage cities from implementing local abatement strategies. The aim of this study was to investigate how critical the choice of ERF is when evaluating traffic-related abatement strategies. We studied this by comparing the potential health benefits of three viable abatement strategies for road traffic in Stockholm and Gothenburg. We also provided an example of how an HIA can be designed that uses different risk estimates for PM_2.5_ originating from local sources and long-range transport, as well as from exhaust and non-exhaust traffic emissions.

## 2. Materials and Methods

### 2.1. Population Exposure

Population exposure to PM in ambient air was estimated in Stockholm and Gothenburg, the largest and the second largest cities in Sweden, respectively. The contributions to PM_2.5_ from local source categories were described using high/resolution Gaussian dispersion modeling, while the contribution from long-range transport (LRT) was estimated based on monitoring data. Near roads, concentrations were modeled with a resolution down to 50 × 50 m^2^. Dispersion from more diffuse sources and at a longer distance was described at lower resolution. Further details on the dispersion modeling and emission inventories involved, including evaluation against measurements, can be found in Segersson et al. [14].

**Figure 1:**
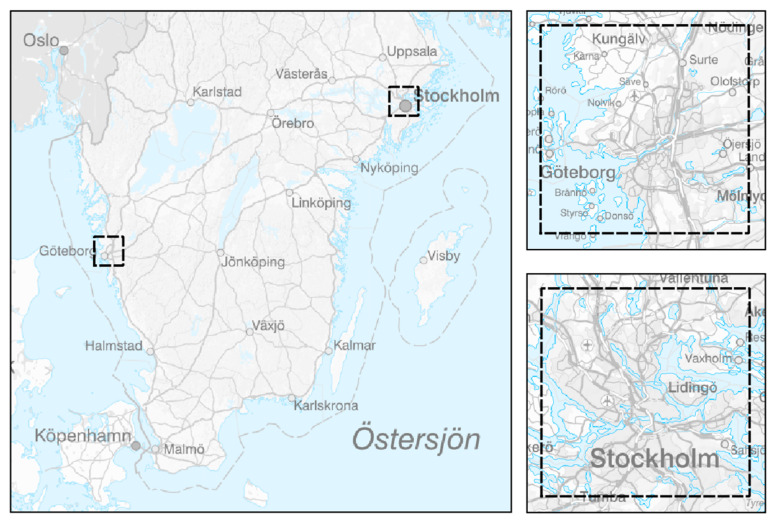
Modeling areas of 35 × 35 km^2^. An overview is displayed on the left; zoomed in maps are shown for Gothenburg (upper right) and Stockholm (lower right).

The current HIA was developed within an area of 35 × 35 km^2^ around the center of each of the 2 cities (see Figure 1). Exposures originating from local sources were divided into the following source categories:

- Residential wood combustion (RWC);
- Road traffic (exhaust);
- Road traffic (wear);
- Shipping;
- Other local sources.

**Figure 2:**
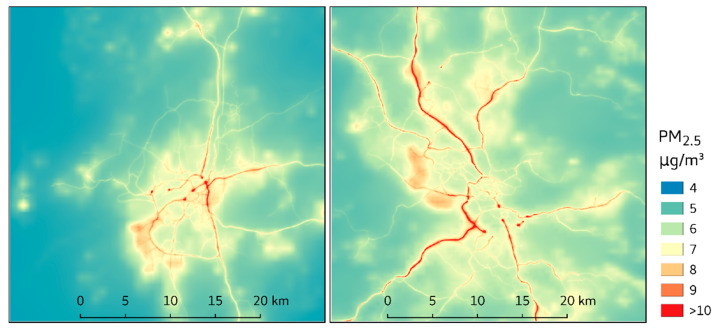
Yearly average concentrations of PM_2.5_ during the baseline year 2011 in Gothenburg (left) and Stockholm (right) [14].

No corresponding division into source categories was made for LRT. As an example, the annual average concentration of PM_2.5_ for 2011, which was used as the baseline for scenario calculations, is presented in Figure 2. Population-weighted concentrations are provided per source category in Table 1.

### 2.2. Abatement Strategies

Effects of different abatement strategies were estimated by modifying source-specific contributions to PM concentrations in proportion to the estimated change in emissions. The evaluated abatement strategies consisted of the introduction of congestion charges, the reduced use of studded tires, and the electrification of light vehicles.

#### 2.2.1. Introduction of Congestion Charges

In Stockholm, a congestion charge was introduced in 2007. The system consists of a toll cordon around the inner city. A few months after the introduction, the traffic reduction across the cordon stabilized at 20–22% [15]. In Gothenburg a similar cordon-based congestion charge was introduced in 2013. The observed traffic reduction across the cordon was 10% on average [16]. For simplicity, the effect of the congestion taxes is represented by an overall reduction in traffic flow within the modelling areas of 21% and 10% for Stockholm and Gothenburg, respectively. Both exhaust and wear emissions (road, tire, and brake wear) are considered proportional to the traffic flow. Since the congestion charges in Stockholm were introduced before the baseline year of 2011, the effect of the congestion charges was determined in this case with ex-post estimations. In Table 2, estimated changes in emissions due to the congestion charges and resulting changes in population exposure are presented.

#### 2.2.2. Reduced Use of Studded Tires

Studded tires are used during winter in order to increase the grip between the tires and the road, and thus reduce the number of accidents. The road wear due to the use of studded tires is an important contributor to PM_2.5_ and PM_10_ in cities of Nordic countries [10,17,18]. In general, use of studded winter tires is allowed in Sweden between 1 October and 15 April. However, some cities where PM_10_ levels are high have banned the use of studded tires on a few streets in order to reduce the overall usage in the cities. In Stockholm 3 streets have a ban and in Gothenburg there is a ban on 2 streets. Both cities promote citizens to use non-studded winter tires and a significant reduction in the share of studded winter tires can be seen.

In Hornsgatan, Stockholm, the maximum share of vehicles using studded tires was reduced from 70% to 36% immediately after the ban and further to below 30% in later years. The combined effect of all emission sources and meteorological factors showed a reduction of around 50% in the street PM_10_ concentration increment averaged for October to May over the years 2011–2014 with the ban in place [18]. If considering the entire year, the reduction would be smaller. To account for this, we assumed that the local road wear emissions over the whole year were reduced by 35% due to studded tire restrictions in both Stockholm and Gothenburg. The road wear was assumed to constitute 75% of the total traffic related wear (road, brake, and tire) and to mainly (around 90%) consist of particles with a diameter >2.5 μm [17]. The estimated changes in emissions and resulting change in population exposure are presented in Table 3.

#### 2.2.3. Electrification of Light Vehicles

Many countries have proposed ambitious policies to reduce greenhouse gas emissions by encouraging a transformation towards a fossil-free vehicle fleet. Stockholm city has set a target to be completely fossil-free by 2040. The plans also include implementation of local environmental zones from which vehicles running on fossil fuels will be banned [19]. We represented this strategy by assuming that 50% of all light vehicles within the modelling area are electrified. The same strategy was also applied in Gothenburg. Exhaust emissions are obviously reduced to zero for electric vehicles, but non-exhaust particles from road, tire, and brake wear are also expected to change. This has been overlooked in some previous studies [20,21], leading to overly optimistic expectations on air quality improvements due to electrification. While particle emissions from brake wear are expected to decrease due to use of regenerative braking systems, road and tire wear emissions are likely to increase due to the higher weight of the electric vehicles. The road and tire wear emissions are assumed proportional to weight [22]. A new electric vehicle is expected to weigh 25% more than a corresponding vehicle with a combustion engine [23]. A reduction by 30% is assumed for brake wear emissions [23]. In countries with cold winters, the increase of road wear is also affected by the use of studded tires. In this scenario, the fraction of electric vehicles using studded tires was the same as for light vehicles in the baseline fleet. Brake wear is assumed to contribute 20% of PM_2.5_ wear emissions and 1% of PM_2.5–10_ wear emissions; the rest of the wear emissions are caused by road and tire wear [17]. Assumptions related to change in wear emissions are summarized in Table 4 and the changes in emissions and population exposure are presented in Table 5.

### 2.3. Health Impact Analysis

**Figure 3:**
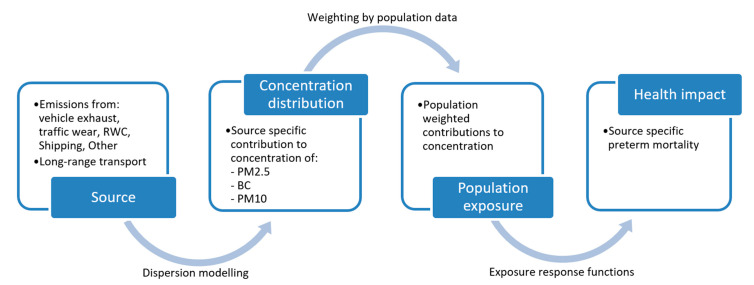
Health impact assessment following the impact pathway chain.

The evaluation of health impact follows the impact pathway chain [24] presented in Figure 3. Using source-specific exposure estimates, the number of premature deaths was attributed to different emission sectors (see Table 1).

Calculations were performed for the baseline in 2011 as well as for the 3 abatement strategies. Monte Carlo simulations were used to estimate the uncertainty range of number of premature deaths (see supplementary S1 for source code). When aggregating uncertainties related to the different sources, the different ERFs were assumed to be independent. The margin of error for exposure estimates was assumed to be 20%. The baseline mortality for natural deaths representing the baseline year was acquired from the Swedish Cause of Death Register at The National Board of Health and Welfare.

In addition to the ERF for PM_2.5_ and mortality recommended by WHO, there is a growing number of alternative assumptions. Data from the very large American Cancer Society’s (ACS) Cancer Prevention Study II (CPS-II) have been used in many influential studies of PM_2.5_ concentrations and mortality, where most studies have used monitor data and described associations of metropolitan-level air pollution (“between-city contrasts”) and mortality [25,26]. In those studies, for many years dominating assessments of PM_2.5_ effects, exposure data were derived at the metropolitan scale, relying on central monitor data. The increase HR in natural mortality using between-city contrasts in PM_2.5_ has been estimated around 1.06 % per 10 µgm^−3^. However, Jerrett et al. [27] also used the ACS CPS-II data but constructed small-area exposure measures (using zip-code) in Los Angeles, California, by interpolation from 23 PM_2.5_ monitors and observed effects nearly 3 times greater than in the models relying on comparisons between communities.

Turner et al. [28] studied 669,046 participants from the ACS Cancer Prevention Study CPS -II with PM_2.5_ concentrations estimated using a national-level hybrid land use regression and Bayesian maximum entropy interpolation model. Estimates of PM_2.5_ were decomposed into near-source and regional components. Ozone and nitrogen dioxide concentrations were also modeled and included in the analyses. In the multi-pollutant model, the hazard ratio (HR) per 10 µgm^−3^ for regional PM_2.5_ became 1.04 (1.02–1.06), whereas for near-source PM_2.5_ it was 1.26 (1.19–1.34).

In a cohort of 635,539 individuals from the US National Health Interview Survey (NHIS), Lefler et al. [29] studied whether the PM_2.5_–mortality relationship differs according to scale of spatial variability. Modeled air pollution exposure estimates for PM_2.5_, other criteria air pollutants, and spatial decompositions (<1 km, 1–10 km, 10–100 km, >100 km) of PM_2.5_ were assigned at the census tract-level. PM_2.5_ mass was largely composed of regional and mid-range components, likely most secondary particles, while the neighborhood and local components contributed a relatively small fraction of PM_2.5_ (23%). The PM_2.5_–mortality association was observed across all 4 spatial scales of PM_2.5_, with higher but less precisely estimated HRs observed for local (<1 km) and neighborhood (1–10 km) variations, scaled by 10 µgm^−3^ 1.299 (95% CI 1.014–1.664) and 1.279 (95% CI 1.173–1.395), respectively, from a joint model with all 4 scales. In a 2-pollutant model with total PM_2.5_ and PM_2.5–10_, the all-cause mortality HR associated with a 10 μgm^−3^ increase in PM_2.5_ was 1.12 (95% CI 1.09–1.15), whereas the HR associated with a 10 μgm^−3^ increase in PM_2.5–10_ was 1.02 (95% CI 1.00–1.04). In the most complex model with total PM levels (with no decompositions), the HR for an IQR increase in PM_2.5_ (3.12) was 1.045 (95% CI 1.030–1.061) and in PM_2.5–10_ it was 1.025 (95% CI 1.011–1.038) per IQR (5.43). This corresponds for the coarse fraction to 1.05 (95% CI 1.02–1.07) per 10 µgm^−3^, and for PM_2.5_ to 1.15 per 10 µgm^−3^.

Spatial variation in particle levels within urban areas is commonly caused by local traffic emissions. When a meta-regression technique was used to investigate the heterogeneity between the studies and whether the study population or analytic characteristics modified the association between PM_2.5_ and mortality, Vodonos et al. [30] found that geographical locations with higher percent of PM_2.5_ coming from traffic were significantly associated with higher estimates with and an HR 1.0205 (95% CI 1.0189–1.0181) per μgm^−3^.

Published reviews often present quantitative summaries of effect size as estimated across studies regardless of the many differences in exposure levels and exposure assessment methods. However, the meta-regression technique used by Vodonos et al. [30] described based on 53 studies with 135 estimated how the PM_2.5_ coefficient decreased in a manner inversely proportional to the mean concentration, and when restricted to studies with mean concentrations below 10 μg/m^3^, the meta-regression estimated an HR of 1.024 (95% CI 1.008–1.04) per 1 μgm^−3^. Less error-prone exposure assessments and greater control for socioeconomic status were also factors associated with larger effect size estimates. Non-linear ERFs which level off at high concentrations have also been suggested when examining the shape of the association between PM_2.5_ and non-accidental mortality applied in the Global Burden of Disease Study [31]. The resulting Global Exposure Mortality Model (GEMM) builds on data from 41 cohorts from 16 countries but does not consider differences between exposure measures or particle sources.

It is becoming more and more apparent that the risk increase per µgm^−3^ PM_2.5_ is greater in areas with low total PM_2.5_ concentrations, for local source contribution, and for traffic emissions than for regional PM_2.5_. Meta-regressions showed that studies with more accurate exposure assessment methods reported larger effect size estimates for PM_2.5_ [32]. Furthermore, within large cohorts the scale of spatial variability in concentrations is important for estimated mortality HRs. Turner et al. [28] observed a more than six times higher HR per absolute increase in concentration for near-source PM_2.5_ in comparison with regional PM_2.5_. Lefler et al. [29] found similar patterns and concluded that regressions using spatially decomposed PM_2.5_ suggest that more spatially variable components of PM_2.5_ may be more toxic. Traffic [30] and low PM_2.5_ exposure [30,32] are factors associated with high HRs. The HR estimated for PM_2.5_ and natural mortality in the Swedish SCAC study [33] falls in the same range as reported by others with exposure variability driven by local sources and is based on the same exposure modeling as this health impact analysis (HIA). Many studies have concluded that there is no evidence of a threshold and that no safe level of PM can be determined [4]. Some assessments have included a threshold to reflect insufficient data at low total concentrations. For this reason, a cut-off of 2 μgm**^−3^** for LRT PM_2.5_ was applied, corresponding to the lowest exposure level with significant associations [5], whereas no cut-off was applied for the anthropogenic local contributions.

Based on the above-mentioned literature, 3 different approaches for the HIA are compared:

- WHO standard
  The same 1.08 (CI 95% 1.06–1.09) HR [32] is applied for PM_2.5_ regardless of source and origin of the particles;
- Separation by distance to source
  HRs based on near-source and regional decompositions [28] are applied to local contributions and LRT, respectively;
- Separation by source category and distance
  Same approach as B., but using an HR for black carbon (BC) [33] to represent vehicle exhaust PM and an HR for PM_10_ [33] to represent vehicle wear PM.

**Figure 4:**
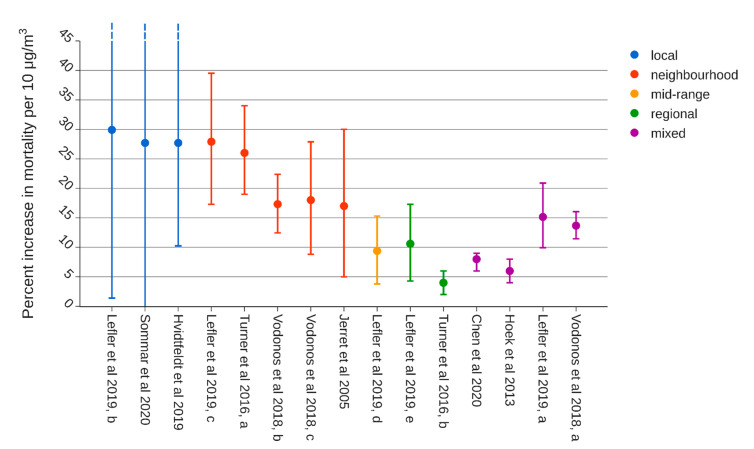
Percent increase in mortality per 10 µgm−3 PM_2.5_ categorized by spatial resolution of exposure data. “Local” corresponds to <1 km resolution, “neighborhood” to 1–10 km, “mid-range” to 10–100 km, and “regional” to >100 km. Confidence intervals (95%) are shown as dashed where outside the scale. In the figure, the estimate denoted as “near-source” by Turner et al. [28] is categorized as the neighborhood scale. The data presented in the figure can be found in Table A1.

In approach B, different HRs are applied for near-source and long-range (regional) contributions to PM_2.5_. In Figure 4, HRs from previously mentioned studies have been labeled according to the spatial resolution of the exposure data on which they are based, showing a clear tendency of higher risk estimates for within-city contributions to PM_2.5_. An overview of the different HRs can be found in Table A1. The study by Sommar et al. [33] is based on the same exposure data as in this study. However, Turner et al. [28] reported an HR for near-source PM_2.5_ with a very similar value but a smaller uncertainty range and presented an HR for regional PM_2.5_. Furthermore, the HR from Turner et al. was adjusted for NO2 and ozone. Therefore, the mortality HRs 1.26 (CI 95% 1.19–1.34) and 1.04 (CI 95% 1.02–1.06) reported by Turner et al. per 10 μgm^−3^ were used for the local and regional contribution to PM_2.5_, respectively.

Since only PM_2.5_ was used as an indicator in approach A and B, changes in coarse PM (larger than 2.5 μm) were not reflected at all in the calculated health effects. This is problematic when evaluating abatement scenarios including significantly larger changes in exposure to coarse PM than to PM_2.5_. In approach C, coarse PM was also included when estimating the impact by using PM_10_ as an indicator for road wear PM with a HR by Sommar et al. [33]. Furthermore, in this approach BC was used instead of PM_2.5_ to estimate health effects related to vehicle exhaust. Since local emissions from vehicle exhaust are an important, often dominating, source of BC in urban areas [34], this choice of indicator is more specific for vehicle exhaust than using HR based on bulk PM_2.5_. The study by Sommar et al. [33] was used since it includes the same geographical areas as this assessment and is based on exposure data with “within-city” contrasts resolved. For other sources of PM than road traffic, the same HR as in approach B was used.

## 3. Results

### 3.1. Evaluation of Abatement Strategies

**Figure 5:**
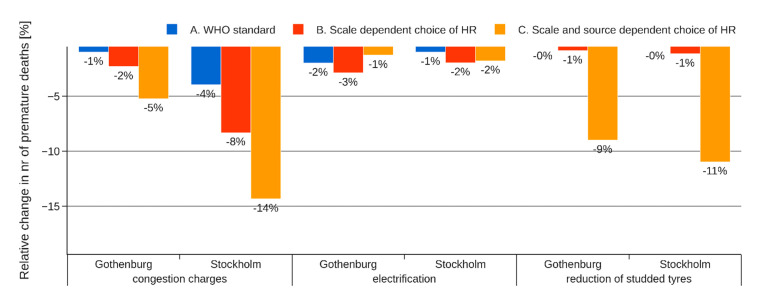
Relative change in mortality attributed to traffic air pollution for the 3 abatement strategies applied in Gothenburg and Stockholm, using 3 different HIA approaches.

Relative changes in number of premature deaths due to traffic air pollution for the three abatement strategies are presented in Figure 5. According to approach A (WHO standard), all three abatement strategies have low potentials in reducing mortality related to air pollution by at most 2%. When considering differences in HR for near-source and long-range contributions to PM_2.5_ (approach B), the associated mortality is reduced by up to 8%. When choosing indicators more representative for traffic-related air pollution (approach C), the associated mortality is reduced by up to 14%. Evidently, the choice of ERF is a critical factor when evaluating local abatement strategies.

For the introduction of congestion charges, the differences between Stockholm and Gothenburg seen for all three HIA approaches in Figure 5 are mainly caused by differences in the observed traffic reduction in the two cities. The differences for the other two abatement strategies are caused by minor differences in the baseline related to vehicle fleet composition, share of PM_2.5_ in wear PM, and share of studded tires.

### 3.2. Source-Specific Change in Mortality

**Figure 6:**
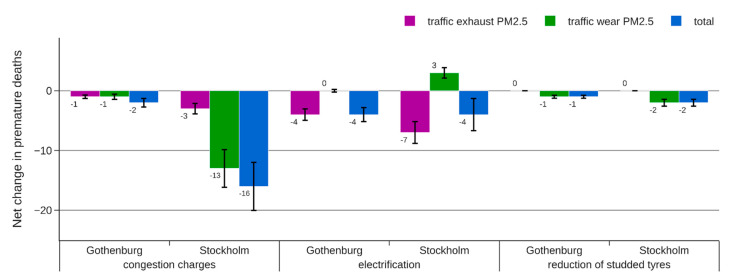
Estimated change in number of premature deaths for each of the abatement strategies following approach A (“WHO standard”). The error bars represent a 95% CI.

**Figure 7:**
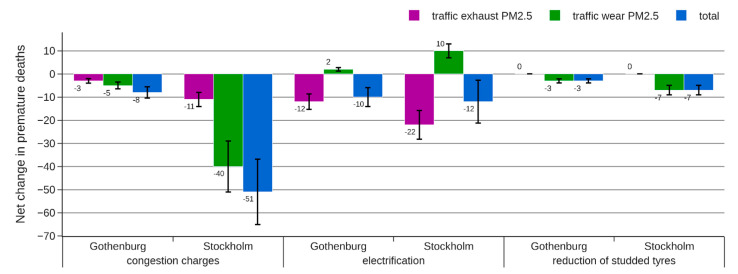
Estimated change in number of premature deaths for each of the abatement strategies following approach B (separation by distance to source). The error bars represent a 95% CI.

**Figure 8:**
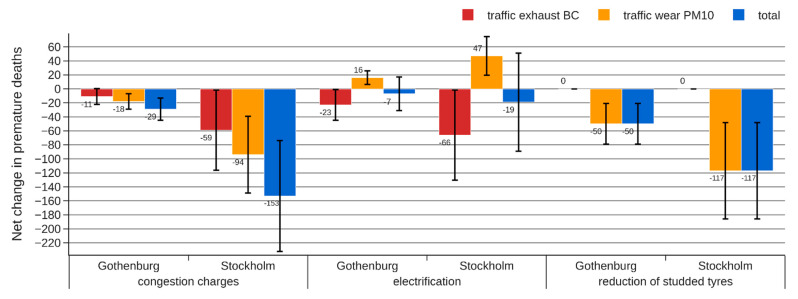
Estimated change in number of premature deaths for each of the abatement strategies following approach C (separation by source category and distance). The error bars represent a 95% CI.

The absolute change in the attributed mortality is presented separately for different sources in Figure 6, Figure 7 and Figure 8.

For the electrification strategy, the increased road wear emissions to some extent compensate for the reduced exhaust emissions, resulting in a modest net reduction in attributed mortality. All HIA approaches indicate an effect in the range of −4 to −19 premature deaths, but the 95% CI of approach C is wider, indicating a possible increase in attributed mortality when the effect of coarse PM is considered in the analysis.

For a reduction of studded tires, approach A (“WHO standard”) and approach B (“Separation by distance to source”) indicate a very small reduction in air pollution-related mortality. The reason is that PM from road wear with a diameter >2.5 μm is not included in the indicator used in these approaches. Since around 90% of wear PM in Stockholm and Gothenburg has a diameter >2.5 μm, there is a risk of almost completely missing any health benefits from this abatement strategy using approach A or B. Approach C instead uses PM_10_ as an indicator for this source resulting in an estimated reduction in premature deaths that is 17–59 times greater.

The introduction of congestion charges reduces both exhaust and non-exhaust emissions and affects all vehicle types, resulting in significant health benefits according to all three HIA approaches. However, the change in attributed mortality is very different for the three HIA approaches, resulting in −16, −51, and −153 premature deaths for approaches A, B, and C, respectively.

## 4. Discussion

The optimal situation for a HIA would be to apply an ERF which is estimated using the same sources, exposure measures, and population as the impact assessment concerns. This suggests that recent studies from the same region are of special value, at least if they are based on a large cohort and offer precise estimates. In real situations it may be realistic to use the most relevant studies with respect to sources and exposure variables.

Long-range transported PM_2.5_ represents around 60–70% of the urban background PM_2.5_ concentration in the largest Swedish cities, but is likely not that dominant when it comes to health impacts. We suggest using a different ERF for near-source PM_2.5_ than for LRT. This is supported by many observations of within-city variations in concentrations (local sources), with traffic emissions and low exposure levels being associated with steeper ERFs. The generally higher HRs observed in populations with low concentrations could likely be explained by a low regional background concentration showing small geographic variability and thus a larger proportion of the variation in exposure explained by local sources.

Non-exhaust emissions are expected to constitute a more and more dominant source of traffic-generated PM_2.5_ in the future [35]. Even though regenerative braking in electric vehicles may reduce brake wear emissions, tire and road wear are likely to increase due to the heavier weight of the electric vehicles that are becoming more common. We assume the same ERF for exhaust and non-exhaust PM_2.5_. Many epidemiological studies make use of PM exposure data based on monitoring data or land-use regression (LUR) [36]. These methods do not in general allow source attribution, making it impossible to distinguish non-exhaust from exhaust PM. While dispersion modeling does allow source attribution, exhaust and non-exhaust PM are highly correlated, making it challenging to separate observed health effects from the two sources statistically. If traffic wear particles in the fine fraction correspond to a smaller HR than those estimated for local sources totally, then the HR for PM_2.5_ from combustion (vehicle exhaust) must be higher than the estimated HR. This has not been shown, and the results from studies of sources and components are not very consistent [37]. However, in Sweden the association with daily mortality has been stronger for road dust and the coarse fraction than for fine PM, NO_2_, and particle number concentration [38,39].

There are several possible explanations as to why near-source PM_2.5_ results in steeper ERFs. Near-source PM_2.5_ contains a larger fraction of particles from traffic and local combustion, while LRT includes more secondary aerosols and particles from natural sources such as sea salt. The LRT aerosol has also spent a longer time in the atmosphere, with aging processes [40] modifying the particles properties. The oxidative potential of PM has been suggested as one of several possible drivers of acute health effects of PM, although the link remains uncertain [41]. PM from anthropogenic sources typically has higher oxidative potential in comparison to secondary aerosols and the crustal material that often dominates the total PM mass concentration [41]. Spatial contrasts within a city or a county are much stronger in near-source PM_2.5_ exposure than in LRT PM_2.5_ exposure. Not resolving these spatial contrasts leads to misclassification of exposure, especially for the most (and least) exposed individuals, which is likely to affect epidemiological analysis. There are probably also differences in the ability to control for confounding between studies with ERFs based on within-city (near-source) and between-city (LRT) exposure contrasts. Epidemiological studies for near-source exposure may be carried out within relatively small geographical areas, thereby reducing the risk of variations in the population and the relevance of covariates adjusted for in the analysis.

We only applied a low threshold for LRT PM_2.5_ in our calculations. The existence of a low threshold has become less and less supported (e.g., the EEA presented mortality impact estimates for PM_2.5_ without using any threshold). In the review for the WHO AQG update, an analysis restricted to studies with a mean exposure below that of the current guideline (10 μgm^−3^) resulted in a steeper combined ERF, and the authors concluded that if a threshold is present, it is at very low levels [32]. It should be noted that for an analysis not using any threshold for LRT PM_2.5_, the total estimated number of premature deaths would have been greater and the relative importance of local sources smaller.

## 5. Conclusions

Among the three evaluated abatement strategies, the largest effect was seen for the reduction of studded tires and using separate ERF for exhaust and non-exhaust sources. However, this was heavily dependent on the choice of ERF applied for coarse PM generated by road wear. The introduction of congestion charges, implying overall traffic reduction, is a safer choice of strategy, and is almost as efficient when health benefits from the reduction of coarse wear PM are considered. This strategy results in a significantly reduced number of premature deaths, except for when the “WHO standard” approach is used, resulting in a reduction of the number of premature deaths within the uncertainty range. The electrification of light vehicles showed a surprisingly low potential to reduce premature mortality, resulting in a small or even insignificant reduction in attributed mortality depending on the choice of ERF.

When estimating premature mortality due to PM_2.5_ exposure, we strongly suggest that different ERFs be applied for near-source and long-range PM_2.5_ exposure. The ERF for PM_2.5_ recommended by the WHO [32] is heavily influenced by epidemiological studies based on between-city (long-range) exposure assessments. Applying a single “average” ERF, as recommended by WHO, therefore results in an underestimation of effects from local measures, effectively sending the message that local measures do not matter.

When the same risk ratio is used to describe both vehicle exhaust and traffic wear PM, wear PM dominates the health impact in Nordic cities. Exhaust PM is sometimes assumed to be more toxic than wear PM. Not reflecting this in the analysis results in a bias towards abatement strategies focusing on PM wear particles. It is difficult to separate effects of exhaust and wear PM in epidemiological studies due to the high correlation between the two variables. Furthermore, ERFs for coarse PM based on multi-pollutant models may to some extent include the effect of exhaust PM (and vice versa). If exhaust PM is assumed to be more toxic than wear PM, an ERF for BC is preferable. BC is not as “diluted” by wear particles and more dominated by vehicle exhaust, making it more representative for this source.

Using HIA to support decisions regarding abatement strategies in cities requires careful consideration regarding the choice of ERF. Applying a single average ERF for different source categories may be misleading in the same way as for near-source and long-range PM_2.5_ exposure. Future epidemiological studies based on source specific exposure estimates can potentially allow the separation of health effects from exhaust and non-exhaust PM as well as from other important urban emission sources.

## Figures and Tables

**Table 1:** Population-weighted concentrations for the baseline year 2011 and population aged > 30 years [14].

Source Category	Population-Weighted Concentration (μgm^−3^)	
	Gothenburg	Stockholm
LRT	4.15	4.60
Vehicle exhaust PM_2.5_	0.27	0.21
Vehicle exhaust PM_10_	0.27	0.21
Vehicle exhaust BC	0.23	0.28
Traffic wear PM_2.5_	0.41	0.73
Traffic wear PM_10_	2.06	2.43
RWC	1.33	0.96
Shipping	0.04	0.02
Other	0.33	0.03

LRT: long-range transport; RWC: Residential wood combustion; BC: black carbon.

**Table 2:** Estimated changes in population-weighted concentrations due to introduction of congestion charges. For Stockholm, the ex-post change is given, i.e., assuming the congestion charge in Stockholm had not been introduced before the baseline year. In the table header, “% of total” refers to total concentration in ambient air.

Emissions	Gothenburg		Stockholm	
All Traffic-Related Emissions	−10%		−21%	
Populated-Weighted Concentration	μgm^−3^	% of Total	μgm^−3^	% of Total
Vehicle exhaust PM_2.5_	−0.027	−0.4	−0.043	−0.7
Vehicle exhaust BC	−0.023	−3.3	−0.058	−8.3
Traffic wear PM_2.5_	−0.041	−0.6	−0.15	−2.3
Traffic wear PM_10_	−0.21	−1.4	−0.51	−3.7

**Table 3:** Estimated changes relative to baseline due to reduced use of studded tires. In the table header, “% of total” refers to the total concentration in ambient air.

Emissions	Gothenburg		Stockholm	
Road Wear PM_10_	−35%		−35%	
Population-Weighted Concentration	μgm^−3^	% of Total	μgm^−3^	% of Total
Traffic wear PM_2.5_	−0.024	−0.37%	−0.028	−0.43%
Traffic wear PM_10_	−0.54	−3.6%	−0.64	−4.6%

**Table 4:** Assumptions and estimations made to calculate change in emissions for a scenario with 50% electrification of light vehicles.

Assumptions	Gothenburg	Stockholm
Share of wear PM_10_ emissions related to light vehicles [14]	75%	91%
Share of exhaust PM_2.5_ emissions related to light vehicles [14]	68%	83%
Assumed share of light vehicles electrified	50%	
Average light vehicle weight increase due to electrification [23]	25%	
Estimated change in total wear PM_2.5_ related to brakes ^1^	20%	
Estimated change in total wear PM_10_ related to brakes ^1^	2%	
Resulting change in road and tire wear PM emissions	+25%	
Resulting change in brake wear PM emissions	−30%	

^1.^ Calculated based on emission factors in NORTRIP [17].

**Table 5:** Estimated changes relative to baseline due to electrification of 50% of light vehicles.

Emissions	Gothenburg		Stockholm	
Vehicle exhaust PM_2.5_	−34%		−42%	
Vehicle exhaust BC	−20%		−24%	
Traffic wear PM_2.5_	4.8%		5.8%	
Traffic wear PM_10_	8.9%		11%	
**Population-Weighted Concentrations**	**μgm^−3^**	**% of Total**	**μgm^−3^**	**% of Total**
Vehicle exhaust PM_2.5_	−0.092	−1.4	−0.086	−1.3
Vehicle exhaust BC	−0.045	−6.4	−0.065	−9.3
Traffic wear PM_2.5_	0.020	0.3	0.042	0.65
Traffic wear PM_10_	0.18	1.2	0.26	1.9

## Data Availability

Data is contained within the article or supplementary material.

## Supplementary Materials

The following are available online at https://www.mdpi.com/article/10.3390/ijerph18136847/s1, Source code and data for HIA S1.

## Appendix A

**Table A1:** HR for all-cause mortality per 10 μgm^−3^ PM_2.5_ categorized according to spatial resolution of the exposure data. “Local scale” represents spatial resolution <1 km, “neighborhood” 1–10 km, mid-range 10–100 km, and “regional” >100 km.

Reference	Spatial Scale	HR
Chen et al. [32]	mixed	1.08 (1.06–1.09)
Hoek et al. [5]	mixed	1.06 (1.04–1.08)
Lefler et al. [29], a	mixed	1.15 (1.10–1.21)
Lefler et al. [29], b	local	1.30 (1.10–1.66)
Lefler et al. [29], c	neighborhood	1.28 (1.17–1.40)
Lefler et al. [29], d	mid-range	1.09 (1.04–1.15)
Lefler et al. [29], e	regional	1.11 (1.04–1.17)
Turner et al. [28], a	local	1.26 (1.19–1.34)
Turner et al. [28], b	regional	1.04 (1.02–1.06)
Vodonos et al. [30], a	mixed	1.14 (1.11–1.16) ^1^
Vodonos et al. [30], b	neighborhood	1.17 (1.12–1.22) ^1,2^
Vodonos et al. [30], c	neighborhood	1.18 (1.19–1.28) ^1,3^
Sommar et al. [33]	local	1.28 (−0.83–1.96)
Hvidtfeldt et al. [42]	local	1.28 (1.10–1.46)
Jerret et al. [25]	neighborhood	1.17 (1.05–1.30)

^1.^ at 10 μgm^−3^. ^2.^ representative for space–time hybrid models [30]. ^3.^ representative for exposure estimates using zip-code level monitoring stations [30].
